# *NR3C1* gene methylation and its association with metabolic syndrome in adults

**DOI:** 10.3389/fnut.2026.1805979

**Published:** 2026-06-25

**Authors:** Amanda Sgrancio Olinda, Aline Ribeiro Borçoi, Suzanny Oliveira Mendes, Ivana Alece Arantes Moreno, Tamires Dos Santos Vieira, Bárbara Risse Quaioto, Marcele Lorentz Mattos de Souza, Carlos Henrique Pagani Corrêa, Manuela Schade da Mota, Ana Paula Stofel Fernandes, Ester Ribeiro Cunha, Flavia Vitorino Freitas, Julia de Assis Pinheiro, Joaquim Gasparini dos Santos, Bruna Pereira Sorroche, Lidia Maria Batista Rebolho Arantes, José Cláudio Casali-da-Rocha, Jones B. Graceli, Adriana Madeira Alvares-da-Silva

**Affiliations:** 1 Biotechnology/Renorbio Graduate Program, Federal University of Espirito Santo, Vitoria, Espírito Santo, Brazil; 2 Pharmacy and Nutrition Department, Federal University of Espirito Santo, Alegre, Espirito Santo, Brazil; 3 Graduation School, Federal University of Espirito Santo, Vitoria, Espirito Santo, Brazil; 4 Laboratório de Investigação Médica (LIM) 31, Hospital das Clínicas HCFMUSP, Faculdade de Medicina, Universidade de São Paulo, São Paulo, Brazil; 5 Molecular Oncology Research Center, Barretos Cancer Hospital, São Paulo, Brazil; 6 A.C. Camargo Cancer Center, São Paulo, Brazil; 7 Department of Morphology, Federal University of Espirito Santo, Vitoria, Espirito Santo, Brazil; 8 Animal Science Program, School of Agricultural Sciences, Southern Illinois University, Carbondale, IL, United States

**Keywords:** epigenetics, glucocorticoid receptor, metabolic syndrome, NR3C1 methylation, pyrosequencing

## Abstract

**Purpose:**

Metabolic syndrome (MetS) is a complex, multifactorial, systemic disease characterized by the coexistence of metabolic abnormalities and is clinically defined by the presence of at least three important risk factors: excess abdominal fat, a low serum concentration of high-density lipoprotein cholesterol, high serum triglyceride levels, high blood pressure, and high serum glucose levels. Understanding the role of epigenetics in MetS remains challenging because of the complexity of its multifactorial mechanisms. Thus, this study aimed to evaluate the factors associated with MetS and their association with methylation in the *nuclear receptor subfamily 3 group C member 1 (NR3C1)* gene.

**Methods:**

This cross-sectional study included 353 volunteers who were patients of the primary care service of the Brazilian Public Health System. Socioeconomic status, lifestyle, and health conditions were assessed, along with anthropometric measurements, blood pressure readings, and the collection of blood samples for biochemical and molecular analyses. The methylation levels of the promoter region (1F) of the *NR3C1* gene were specifically analyzed via DNA pyrosequencing.

**Results:**

The prevalence of MetS was 40.5% in the present study, and factors associated with MetS in the multivariate Poisson regression were age, excess body fat, serum v*ery low-density lipoprotein* (VLDL) cholesterol levels, and methylation at C*p*Gs 40 and 46 of the *NR3C1* gene.

**Conclusion:**

In summary, these findings demonstrate a site-specific association between NR3C1 DNA methylation and MetS and highlight a molecular profile that coexists with established clinical risk factors. These results suggest a potential epigenetic role of the NR3C1 gene in MetS, contributing to a better understanding of its etiology.

## 1 Introduction

Most modern lifestyles are based on the consumption of a high-calorie diet characterized by excessive intake of carbohydrates and ultra-processed foods (1). This lifestyle change is relatively new and is closely associated with the emergence of chronic diseases, such as obesity, hypertension, insulin resistance, diabetes and metabolic syndrome, probably as a result of environmental pressure and the need for metabolic adaptation to a high-calorie diet (2).

Metabolic syndrome (MetS) is a complex, multifactorial, systemic disease characterized by the coexistence of metabolic abnormalities and is clinically defined by the presence of at least three important risk factors: excess abdominal fat, a low serum concentration of high-density lipoprotein (HDL) cholesterol, high serum triglyceride (TG) levels, high blood pressure (BP), and high serum glucose levels (3).

MetS has attracted the interest of the scientific community because of the increasing cost of healthcare and the increased risk of metabolic and cardiovascular diseases, such as high BP, hepatic steatosis, stroke, chronic kidney disease, polycystic ovarian syndrome (PCOS) and type 2 diabetes mellitus (4, 5).

MetS affects a large share of adults worldwide and drives major chronic disease and economic burdens. Recent analyses estimate the MetS prevalence in United States adults to be approximately 41.8% in 2017–2018, whereas a pooled analysis of Brazilian studies revealed an overall prevalence of approximately 33% (higher in women than in men) (6, 7). MetS markedly increases the risk of type 2 diabetes, atherosclerotic cardiovascular disease, heart failure and chronic kidney disease and accelerates morbidity and mortality from these and other pathological conditions (8).

Moreover, epigenetic mechanisms, particularly DNA methylation, appear to be among the main factors regulating metabolic adaptation in these abnormalities (9). In this context, studies have reported the involvement of DNA methylation of specific genes, including *NR3C1*, in the onset of diseases, including metabolic disorders (9–12). The *NR3C1* gene encodes the glucocorticoid receptor (GR), a protein essential for regulating Hypothalamic-pituitary-adrenal axis (HPA) and stress responses as well as modulating inflammatory responses that can contribute to chronic metabolic diseases, such as obesity, insulin resistance and MetS. However, epigenetic involvement in the etiology of MetS has not been well elucidated.

Understanding the role of epigenetics in MetS remains challenging because of the complexity of its multifactorial mechanisms. However, studies exploring *NR3C1* methylation in the context of MetS remain scarce, especially in Brazilian populations. Therefore, the present study aimed to identify factors associated with MetS and to verify whether methylation levels in the promoter region (1F) of the *NR3C1* gene are associated with MetS.

## 2 Methods

### 2.1 Ethics statement

In this cross-sectional study, 353 adults aged between 20 and 59 years were recruited from primary care services of Brazilian public healthcare in Alegre-ES, southeast Brazil. Participants were included if they had the ability to answer the questionnaires and had measurements of their waist circumference, HDL cholesterol, TG, BP, and blood glucose. Those without these essential clinical measurements were excluded from the analysis (WC, HDL, TG, BP, or glucose). This study was approved by the Human Research Ethics Committee (CEP CCS/UFES #1.574.160/2016 and #3.420.734/2019). All the volunteers signed an informed consent form.

### 2.2 Socioeconomic status

Data were collected through individual interviews and semistructured questionnaires and divided into two sections: socioeconomic status (age group in years, biological sex, schooling in years, and family income) (13) and lifestyle (physical inactivity/no physical inactivity, smoking habit or no, and alcohol consumption or no) (14). The family income variable was categorized as low income if its per capita income per day was less than $5 (five US dollars) (14).

### 2.3 Anthropometric measurements

Anthropometric measurements, including height (cm), weight (kg), waist circumference (WC, cm), body fat percentage (BF, %), and body mass index (BMI) (14), were obtained by trained professionals in the morning between 7:00 and 8:00 a.m., following an eight-hour fast, in accordance with the technical standards of the Food and Nutrition Surveillance System. Patients were instructed to wear minimal clothing, remove their shoes, stand upright so that their feet were in contact with the metal part of the scale, extend their arms around their body, align their head aligned with their posture and gaze fixed at a fixed point (15, 16).

The height was measured via the Alturexata® stadiometer, which has a maximum capacity of 2.10 meters. Weight was measured via a Tanita® bipolar bioimpedance scale, which was connected to a BC601® body fat monitor. The capacity of the scale ranged from 100 g to 150 kg. Waist circumference was measured via nonstretchable anthropometric tape, with the midpoint between the last rib and the iliac crest as the reference point. The classification of body fat percentage was conducted in accordance with age group and sex (14). Body fat excess was adjusted for sex and age. Excess body fat was defined as >34% for women aged 18–39 and >35% for those aged 40–59; and for men, >21% (18–39 years) and >23% (40–59 years) (16).

Body mass index (BMI) was calculated and classified according to the reference values of the World Health Organization (2000), where individuals with a BMI < 18.5 were considered underweight, those with a BMI ≥ 18.5 and <25 were classified as having normal weight, those with a BMI ≥ 25 and <30 were classified as overweight, and those with a BMI > 30 were classified as obese. A dichotomous categorization of “overweight” and “nonoverweight” was determined using cutoff values of BMI ≥ 25 and BMI < 25, respectively. Body fat percentage was classified according to age and sex and further categorized into “non-overweight” and “overweight” (16).

Systolic blood pressure (SBP, mmHg) and diastolic blood pressure (DBP, mmHg) were measured via a G-TECH® Premium Aneroid sphygmomanometer after a 5-min rest period. The technique employed to measure blood pressure was in accordance with the recommendations outlined in the VI Brazilian Guidelines on Hypertension of the Brazilian Society of Cardiology (17).

### 2.4 Sample preparation, biochemical analysis, and DNA extraction

Blood samples were collected in the morning between 7:00 a.m. and 8:00 a.m. after a fasting period of at least 12 h. Ten milliliters of blood were obtained via venipuncture via disposable syringes. Peripheral blood was transferred to tubes containing ethylenediaminetetraacetic acid (EDTA) for subsequent biochemical and molecular analysis. DNA was extracted via the salting-out method with saline precipitation (18). The concentration, quality, and quantity of DNA in each sample were determined via a NanoDrop spectrophotometer.

### 2.5 Metabolic syndrome and other biochemical variables

According to the Adult Treatment Panel III (ATP-III) of the National Cholesterol Education Program (NCEP), the metabolic syndrome variable was categorized as altered when the patient had three or more altered risk factors (waist circumference, HDL cholesterol, triglycerides, blood pressure, and blood glucose).

The cut-off point considered in the study was: waist circumference >102 cm (men), > 88 cm (women); High-Density Lipoprotein (HDL) cholesterol <40 mg/dL (men) <50 mg/dL (women); blood pressure ≥130 mmHg or ≥85 mmHg for both; triglycerides ≥150 mg/dL and blood glucose ≥100 mg/dL (19, 20).

For categorizing low-density lipoprotein (LDL), reference values that were <100 were considered normal, and values >100 were considered altered. Very low-density lipoprotein (VLDL) < 40 mg/dL was considered normal, and values >40 mg/dL were considered altered. Total cholesterol was considered normal at a value <200 mg/dL and altered at a value >200 mg/dL, according to the V Brazilian Guideline on Dyslipidemia and Prevention of Atherosclerosis. These variables were not considered risk factors for categorization into metabolic syndrome but were used as confounding factors in the statistical analyses.

### 2.6 Quantitative pyrosequencing methylation assays

The region of interest for the *NR3C1* gene corresponds to the 1F region, spanning positions 961 to 1,371 (GenBank: AY436590.1, NCBI). The analyzed CpG sites corresponded to CpGs 40–47, as described by Palma-Guidel et al. (21) and Borçoi et al. (22), and to CpG sites 6–13, according to Oberlander et al. (23). The selection of CpGs 40–47 in the *NR3C1* 1F region was based on prior functional studies indicating that these loci are sensitive to environmental stress and modulate glucocorticoid receptor transcriptional activity (21, 23, 24). This region has also shown stress-related methylation variability in metabolic and psychiatric contexts (24).

One microgram of genomic DNA extracted from each patient was treated with bisulfite via the EZ DNA Methylation™ Kit (Zymo Research) following the manufacturer’s instructions. For the polymerase chain reaction, 1 μg of extracted and converted (bisulfite-treated) DNA was used. The HotStart Taq DNA Polymerase (Qiagen®) and primers were adapted from previous studies (23, 25). To confirm the quality of the PCR product and the lack of contamination, an agarose gel (2%) was run using GelRedTM (Uniscience®).

The PCR products were sequenced via a PSQ96ID Pyrosequencer (Qiagen, Valencia, CA, United States) with a PyroMark Gold Q96 Reagent Kit (Qiagen, Valencia, CA) according to the manufacturer’s protocol (26). Among the 353 participants included in the study, only 294 had sufficient DNA for the analysis of the methylation levels of the *NR3C1* gene. All the conditions for the PCR, pyrosequencing, and sequencing primers are listed in Table 1.

**Table 1:** Primer*s* and PCR conditions for amplifying the 1F region of the *NR3C1* gene.

Primers *NR3C1*		PCR conditions		
Forward	5′-TTTTTTTTTTGAAGTTTTTTTA-3′	95 °C	(14′30″)	
Reverse	5’-BIOTIN-CCCCCAACTCCCCAAAAA-3′	94 °C 50 °C 72 °C	(30″) (30″) (30″)	45x
		72 °C	(10′) indefinitely	
		4 °C		

Sequencing primers	
40 to 42 C*p*G	5′-AGAAAAGAAATTGGAGAAATT-3′
40 to 42 C*p*G	5′-GTTTTAGAGAGATTAGGT-3′
Analyzed sequences	
Seq 1	YGGTGGTTTTTTTAAYGTYGTTTTAATCGTGTTGATCAGTCGCTTA
Seq 2	YGGTTTTYGTYGTTGTYGTYGTTAGTCAGTTCAGTCGTAGTCAGTCGTA

NR3C1: nuclear receptor subfamily 3 group C member 1.

### 2.7 Statistical analysis

The Shapiro–Wilk test was used to verify normality. Categorical data are presented in tables with relative and absolute frequencies and were compared via the chi-square test at a significance level of 5% (<0.05). Univariate and multivariate analyses were performed to identify the factors associated with MetS, using a Poisson regression model with robust variance to account for potential overdispersion via the backward method: variables with *p* < 0.20 in the univariate analysis were included in the initial multivariate Poisson regression. Model refinement was then conducted via a manual backward elimination process, where a *p*-value <0.05 was required for a variable to be retained in the final model. All the statistical analyses were performed via IBM SPSS Statistics version 20 and STATA/MP version 14.1.

## 3 Results

### 3.1 Socioeconomic, lifestyle and anthropometric characterization of the sample

Among the users Brazilian Public Unified Health System, data were collected from 353 participants who comprised the sample of this study. Of them, 285 (80.7%) were women and 68 (19.3%) were men. Most of the participants belonged to the 40 to 59 age group, representing 59.2% of the total sample, and had an income of ≤1 minimum wage (67.8%). Furthermore, of the 353 participants, 243 (68.8%) were overweight, 233 (67.3%) had excess body fat, and 143 (40.5%) met the criteria for MetS. An important association between age and education level and MetS was also detected (*p* < 0.001 and *p* = 0.031, respectively). The other characteristics that were not significantly associated with MetS are shown in Tables 2, 3.

**Table 2:** Socioeconomic and lifestyle characterization of the sample according to metabolic syndrome parameters.

Characteristics	Metabolic syndrome						p
	Total		Yes		No		
	*N*	(%)	*N*	(%)	*N*	(%)	
Age range (years old)							
20 to 39	144	40.8	38	26.6	106	50.5	<0.001*
40 to 59	209	59.2	105	73.4	104	49.5	
Sex							
Female	285	80.7	117	81.8	168	80.0	0.671
Male	68	19.3	26	18.2	42	20.0	
Education							
>8 years old	193	55.9	68	48.9	125	60.7	0.031*
<8 years old	152	44.1	71	51.1	81	39.3	
Not evaluated#	8						
Family income							
>1 minimum wage	111	32.2	46	32.4	65	32.0	0.942
≤1 minimum wage	234	67.8	96	67.6	138	68.0	
Not evaluated#	8						
Smoking habit							
Yes	93	26.4	43	30.1	50	23.9	0.199
No	259	73.6	100	69.9	159	76.1	
Not evaluated#	1						
Alcohol consumption							
Yes	187	53.3	78	54.5	109	52.4	0.639
No	164	46.7	65	45.5	99	47.6	
Not evaluated#	2						
Physical inactivity							
No	216	62.2	90	64.7	126	60.6	0.432
Yes	131	37.8	49	35.3	82	39.4	
Not evaluated	6						

Categorical variables are presented in relative (%) and absolute (*N*) frequencies. #Not available: number of participants not available (not considered in the statistical calculations). Family income: minimum wage corresponds to R$937 or $ 297. *p-value for the chi-square test. With a significance level of ≤5%.

**Table 3:** Anthropometric characterization according to metabolic syndrome parameters.

Characteristics	Total		Metabolic syndrome				*p*
			Yes		No		
	*N*	(%)	*N*	(%)	*N*	(%)	
Body mass index							
Overweight	243	68.8	124	86.7	119	56.7	<0.001*
No overweight	110	31.2	19	13.3	91	43.3	
Body fat							
Excess body fat	233	67.3	121	87.7	112	53.8	<0.001*
No excess body fat	113	32.7	17	12.3	96	46.2	
Not evaluated#	7						
Blood pressure							
Normal	222	63.1	42	29.4	180	86.1	<0.001*
Abnormal	130	36.9	101	70.6	29	13.9	
Waist circumference							
Normal	165	46.7	22	15.4	143	68.1	<0.001*
Abnormal	188	53.3	121	84.6	67	31.9	

Categorical variables are presented in relative (%) and absolute (*N*) frequencies. #Not available: number of participants not available (not considered in the statistical calculations). * *p*-value for the chi-square test. With a significance level of ≤5%.

### 3.2 Characterization of the sample’s biochemical parameters

The lipid profile revealed elevated total blood cholesterol levels in 32%, LDL levels in 34.6% and VLDL levels in 19.5% of the patients. All the variables used to characterize the biochemical parameters were associated with MetS (Table 4). A significant association was observed between MetS and most biochemical parameters, with the exception of HDL cholesterol (*p* = 0.068) and the LDL/HDL ratio (*p* = 0.935).

**Table 4:** Characterization of biochemical parameters.

Characteristics	Total		Metabolic syndrome				*p*
			Yes		No		
	*N*	(%)	*N*	(%)	*N*	(%)	
Total cholesterol							
Normal	240	68.0	84	58.7	156	74.3	0.002*
Abnormal	113	32.0	59	41.3	54	24.7	
LDL-C							
Normal	231	65.4	83	58.0	148	70.5	0.016*
Abnormal	122	34.6	60	42.0	62	29.5	
VLDL-C							
Normal	284	80.5	93	65.0	191	91.0	<0.001*
Abnormal	69	19.5	50	35.0	19	9.0	
HDL-C							
Normal	85	24.1	27	18.9	58	27.6	0.059
Abnormal	268	75.9	116	81.1	152	72.4	
LDL/HLD							
Normal	299	84.7	121	84.6	178	84.8	0.970
Abnormal	54	15.3	22	15.4	32	15.2	
Triglycerides							
Normal	225	63.7	52	36.4	173	82.4	<0.001*
Abnormal	128	36.3	91	63.6	37	17.6	
TyG index							
Normal	168	48.1	26	18.4	142	68.3	<0.001*
Abnormal	181	51.9	115	81.6	66	31.7	
Glucose							
Normal	241	68.3	54	37.8	187	89.0	<0.001*
Abnormal	112	31.7	89	62.2	23	11.0	

VLDL, very low-density lipoprotein; LDL, low density lipoprotein; HDL-C, high-density lipoprotein cholesterol; TyG, triglyceride-glucose index; LDL/HDL, low-density lipoprotein to high-density lipoprotein ratio. Categorical variables presented as relative (%) and absolute (*N*) frequencies; * *p*-value for the Chi-square test. With a significance level of ≤5%.

### 3.3 Factors associated with MetS

The factors associated with MetS in the univariate Poisson regression (*p* < 0.2) were age group, body fat, VLDL cholesterol, and methylation of C*p*Gs 40 and 46 in the 1F region of the *NR3C1* gene. These factors were used for multivariate Poisson regression analysis with robust variance. According to the model, MetS was explained by age, body fat, VLDL cholesterol levels, and the methylation of C*p*Gs 40 and 46 of the 1F region of the *NR3C1* gene, as shown in Table 5.

**Table 5:** Multivariate poisson regression analysis with robust variance.

Dependent variable: MetS	Multivariate poisson regression	
Independent factors	IRR (95% CI)	*p*
Age range	1.58 (1.16–2.16)	0.003
Body fat	2.85 (1.78–4.55)	<0.001
VLDL-C	1.88 (1.49–2.37)	<0.001
*NR3C1* methylation at C*p*G 40	1.35 (1.00–1.81)	0.048
*NR3C1* methylation at C*p*G 46	1.87 (1.52–2.29)	<0.001

VLDL, very low-density lipoprotein; CpG, cytosine-phosphate-guanine; IRR, prevalence value; CI, confidence interval; *p*-value < 0.05 for poisson multivariate regression with robust variance. With significance of 5% and metabolic syndrome (MetS) as the dependent variable.

The findings of the present study indicate that the prevalence of MetS was 1.58-fold greater among individuals aged 40–59 years. Furthermore, individuals who are overweight and have altered VLDL cholesterol levels demonstrate 2.85-fold and 1.88-fold increases in the prevalence of MetS, respectively.

With respect to the methylation of CpGs 40 and 46, every 1% increase in methylation in these CpGs was associated with a 35 to 87% increase in the prevalence of MetS.

## 4 Discussion

This study evaluated the presence of MetS, factors associated with MetS and their relationship with site-specific DNA methylation in the *NR3C1* gene among adults assisted by the Brazilian public healthcare system. The prevalence of MetS in this population was 40.5%, and multivariate Poisson regression identified age, body fat percentage, VLDL cholesterol levels, and methylation at CpG sites 40 and 46 of the *NR3C1* gene as associated variables. Together, these findings highlight the multifactorial nature of MetS, integrating clinical, biochemical, and molecular components.

The high prevalence of MetS observed (40.5%) aligns with the global epidemiological landscape and is consistent with the prevalence described in previous studies. Such as Oliveira et al. (27), which reported that the prevalence of MetS in the Brazilian adult population is 38.4%. The pooled estimate of the MetS prevalence among subjects in Brazil was determined to be 33% (7). Studies conducted in Mexico and Saudi Arabia reported rates of 36 and 39.8%, respectively (28, 29). In the United States, the prevalence reached 41.8% during the 2017–2018 period (6), suggesting that variation across countries is likely driven by methodological, ethnic, and cultural differences across populations.

In this study, the prevalence of MetS was 2.85 times higher in individuals with excess body fat. This result was expected since obesity is associated with MetS complications (30). These findings indicated a heightened prevalence of MetS, with an observed increase of 1.58 times in the 40–59 age group. This observation has been corroborated by other studies (31, 32).

Furthermore, the prevalence of MetS was 1.88 times higher in individuals with altered VLDL cholesterol levels. This association has been reported previously. Dyslipidemia associated with MetS is characterized by increased triglycerides, reduced HDL-c, a predominance of LDL-c, reduced lipoprotein lipase activity, and a predominance of VLDL-c particles of greater volume and with altered content associated with greater atherogenic capacity, increasing the risk for cardiovascular complications (31–33).

There is a consensus in the literature regarding the genetic and environmental risk factors attributed to MetS, such as polymorphisms, lifestyle, eating habits, health conditions, sedentary behavior, and stress (4, 34, 35). However, owing to its multifactorial complexity, the etiology of MetS has not been fully elucidated (4).

The relationship between the environment and the epigenome has been increasingly explored in the pathophysiology of chronic diseases. Thus, the present study also investigated this potential association. Multivariate Poisson regression analysis with robust variance revealed well-established risk factors for MetS and provides evidence for a site-specific association with the *NR3C1* DNA methylation. The analysis revealed an independent association between higher methylation levels at *NR3C1* CpGs 40 and 46 and MetS. Specifically, for every 1% increase in methylation at CpGs 40 and 46, the prevalence of MetS increased by 35 and 87%, respectively.

The association between *NR3C1* methylation at CpGs 40 and 46 and MetS prevalence provides a site-specific molecular profile that coexists with established risk factors, such as age, dyslipidemia and excess body fat. These findings are consistent with recent state-of-the-art evidence that suggests that the epigenome may act as a dynamic record of lifetime exposures, such as environmental stress and lifestyle factors, reflecting the current metabolic state (36, 37). In this context, site-specific DNA methylation signatures are increasingly recognized as markers that appear in the early stages of chronic metabolic diseases, potentially linking environmental and metabolic pressures to metabolic disorders (12) and metabolic syndrome (38, 39). Thus, these results suggest that this locus may be part of the epigenetic signature associated with chronic metabolic disturbances.

The pathophysiological significance of these findings lies in the role of the *NR3C1* gene as a regulator of the HPA axis. The *NR3C1* 1F promoter region is a recognized stress-sensitive locus characterized by a high concentration of CpG dinucleotides (21, 24, 40). Mechanistic models in the literature suggest that elevated methylation at regulatory regions of *NR3C1* is biologically consistent with reduced transcription and glucocorticoid receptor (GR) sensitivity, a state described as systemic glucocorticoid resistance (40–42). This impaired feedback inhibition is a critical mechanism in metabolic syndrome pathophysiology, as inappropriate glucocorticoid signaling favors visceral fat accumulation, disrupts lipid metabolism, and exacerbates low-grade inflammation (12, 33, 43). However, it is important to note that we did not directly measure GR protein expression or serum cortisol levels in this study.

Emerging evidence indicates that chronic environmental and psychosocial stressors can shape *NR3C1* methylation through sustained activation of the HPA axis. This adaptive response may contribute to long-term alterations in GR sensitivity and metabolic processes (11, 24, 40, 44). Although the current study cannot establish causality, the observed association between CpG-specific methylation and MetS prevalence may align with the concept of the epigenetic embedding of environmental stress, which influences health and well-being.

A dysfunctional GR may be indicative of a dysfunctional HPA axis and altered cortisol and has been associated with an impaired adaptive response to chronic stress. Inappropriate glucocorticoid signaling has been demonstrated to contribute to increased activation of inflammatory responses, psychopathologies, disruption of metabolic processes, accumulation of visceral fat, lipid profiles, insulin resistance, glucose levels, dyslipidemia, and hypertension (13, 22, 40, 44–46). It has been proposed that the low-grade inflammation characteristic of obesity may interact with HPA axis function (33, 38, 43), potentially influencing *NR3C1* methylation patterns, although current evidence remains limited (42, 47). Thus, our unpublished findings regarding CpGs 40 and 46 strengthen the hypothesis that site-specific *NR3C1* methylation serves as a biological interface through which the cumulative impact of environmental and metabolic factors is expressed in the complex phenotype of Metabolic Syndrome.

Genes such as *FKBP prolyl isomerase 5 (FKBP5)*, which are functionally interconnected with *NR3C1* in regulating GR sensitivity, have also demonstrated methylation changes under metabolic conditions (43). Ortiz et al. (48) reported an association between higher levels of glycated hemoglobin and low-density lipoprotein cholesterol, both of which are characteristics of MetS, and intron 2 FKBP5 methylation at one CpG site in 43 adults with type 2 diabetes, supporting the hypothesis that HPA axis–related epigenetic alterations may co-occur in metabolic dysfunction.

Other regulatory pathways, including the adenosine monophosphate-activated protein kinase (AMPK) and peroxisome proliferator-activated receptor (PPAR) and mammalian target of rapamycin (mTOR) signaling, are known to integrate nutritional and energy signals with metabolic processes (10, 12). The mTOR pathway, specifically, functions as a nutrient-sensing mechanism involved in cellular energy balance (49). Evidence indicates that mTORC1 signaling modulates pancreatic beta-cell function and insulin sensitivity, with potential interactions with epigenetic modifications such as DNA methylation (12, 50, 55). While these markers were not quantified in the present study, the association between *NR3C1* methylation and MetS components suggests a molecular network where stress-responsive pathways and nutrient sensors converge in the metabolic disturbances observed in this population.

The epigenetic relationship between the *NR3C1* gene and MetS remains unclear. Studies suggest a potential association between epigenetic mechanisms and environmental/metabolic factors or multifactorial chronic diseases (43, 46, 51). De Assis Pinheiro and collaborators (13) and Freitas et al. (51) verified the association between overweight and obesity with hypomethylation of the same region of the *NR3C1* gene in this study, suggesting the involvement of chronic stress and low-grade inflammation with excess fat, factors closely related to MetS consequences (13, 52). Borçoi et al. (52) analyzed a cohort of obese individuals and reported an association between methylation at the region and the prevalence of physical inactivity. Physical inactivity is a well-established correlate of MetS (35, 51).

Similarly, Souza et al. (48) reported associations between *NR3C1* DNA methylation, abnormal serum glucose levels, and insulin resistance (47). Together, these findings reinforce the biological plausibility of *NR3C1* as a stress-responsive locus involved in metabolic regulation in our current study. Luttmer et al. (53) reported that individuals with two MetS components, particularly hyperglycemia and low HDL cholesterol levels, exhibited global DNA hypomethylation, not gene specific, compared with individuals without metabolic syndrome components (52).

Kolb et al. (54) identified polymorphisms in the *NR3C1* gene that appear to modulate susceptibility to MetS in a genetically isolated population. However, no statistical correlation with epigenetic alterations in this gene was found (51). Womersley et al. (44) proposed that differential methylation of genes associated with the HPA axis is a potential pathway mediating the association with MetS (43). Another possible mechanism associated with MetS methylation of the *NR3C1* gene involves changes in diet and lifestyle, which appear to act as environmental pressures on metabolism-regulating genes, such as the *NR3C1* gene (47).

These findings, together with associations observed in *NR3C1*, underscore the possibility that stress-responsive gene networks contribute to MetS pathophysiology, although longitudinal studies are needed to clarify temporal directionality. This was a cross-sectional study and we cannot establish a causal relationship; that is, we cannot assert whether *NR3C1* gene methylation is a risk factor for the development of metabolic syndrome or if the syndrome itself alters *NR3C1* methylation patterns. Furthermore, DNA methylation was measured at a single time-point, which does not account for the dynamic nature of epigenetic marks that can change over time. Additionally, this study lacks replication cohort and functional validation, such as GR protein expression or cortisol measurements. Therefore, our epigenetic findings reflect statistical associations, and further mechanistic studies are required to strengthen biological interpretation.

Another limitation of the study is the predominance of female subjects in the study population. The MetS was calculated according to sex-specific criteria and no significant differences in MetS were observed between men and women in our sample, as evidenced in the Supplementary Table 1, which reflects that the metabolic risk profiles of the studied population are like those of men. However, we acknowledge that this female predominance may limit the generalizability of our findings to the broader male population.

We also acknowledge the potential for residual confounding by unmeasured variables, such as detailed dietary intake, sleep quality, medication use, inflammatory status, psychological stress levels, and adverse childhood experiences, which are known modulators of the NR3C1 gene and metabolic outcomes. Finally, the molecular analysis was also limited by the exclusion of 16.7% of the initial sample due to insufficient DNA yield or quality. However, a comparative analysis between the participants included in the molecular study (*n* = 294) and those excluded (*n* = 59) revealed no significant differences in a subsample terms of age (*p* = 0.575), sex (*p* = 0.895), or MetS status (*p* = 0.157). This indicates that the data were missing at random, thereby minimizing the risk of selection bias.

Further research is needed to fully understand the modulation that occurs during this association. Exploring the relationship between MetS and epigenetics provides new perspectives for the development of more effective therapeutic and preventive strategies. Thus, this study provides relevant epidemiological data on MetS for government entities and health professionals, providing support for the development of public prevention and treatment policies that contribute to reducing public health spending, since the accelerated growth of this condition is recurrent on a global scale and is directly related to the development of chronic diseases in the population.

## 5 Conclusion

This study provides evidence that MetS is associated with *NR3C1* DNA methylation in adults via Brazil’s public health system. These findings contribute to the understanding of stress-responsive epigenetic mechanisms involved in metabolic regulation. Future longitudinal and mechanistic research is warranted to clarify the temporal sequence and biological mechanisms involved, potentially integrating multiomics data to elucidate how stress-responsive genes modulate metabolic homeostasis.

## Data Availability

The raw data supporting the conclusions of this article will be made available by the authors, without undue reservation.
