# Supplementary material for: NR3C1 gene methylation and its association with metabolic syndrome in adults
Source: Front Nutr. 2026 Jun 25;13:1805979. doi: 10.3389/fnut.2026.1805979 (PMC13345824; doi:10.3389/fnut.2026.1805979)
Supplement: Supplementary file 1 [file Table_1.DOCX]

Supplementary Material

# Supplementary Table

**Table:** Univariate Poisson regression analysis with robust variance.

| **Dependent variable: MetS** | **Univariate Poisson regression** | |
| --- | --- | --- |
| **Independent Factors** | **IRR (CI 95%)** | **p** |
| Sex | 1.07 (0.76 - 1.49) | 0.676 |
| Age range (years old) | 1.90 (1.40 - 2.58) | **<0.001*** |
| Education | 0.75 (0.58 – 0.97) | 0.031 |
| Marital Status | 1.12 (0.86 - 1.47) | 0.384 |
| Family income | 0.98 (0.75 - 1.29) | 0.942 |
| Children | 1.74 (1.14 - 2.66) | **0.009*** |
| Leisure activity | 1.04 (0.81 - 1.35) | 0.716 |
| Physical inactivity | 0.89 (0.68 - 1.17) | 0.437 |
| Alcohol consumption | 1.05 (0.81 - 1.35) | 0.694 |
| Smoking habit | 1.19 (0.91 -1.56) | 0.187 |
| Body Mass Index | 2.95 (1.92 -4.53) | **<0,001*** |
| Body fat | 3.54 (2.18 - 5.44) | **<0,001*** |
| Cortisol | 0.84 (0.51 - 1.37) | 0.492 |
| Total Cholesterol | 1.49 (1.16 - 1.90) | **0.002*** |
| VLDL_c | 2.21 (1.77 - 2.76) | **<0.001*** |
| LDL_c | 1.36 (1.06 - 1.75) | **0.014** |
| *NR3C1* methylation at C*p*G 40 | 1.52 (1.08 - 2.13) | **0.015** |
| *NR3C1* methylation at C*p*G 41 | 0.87 (0.39 - 1.93) | 0.733 |
| *NR3C1* methylation at C*p*G 42 | 0.77 (0.24 - 2.42) | 0.663 |
| *NR3C1* methylation at C*p*G 44 | 2.27 (1.99 - 2.60) | **<0,001*** |
| *NR3C1* methylation at C*p*G 46 | 2.25 (1.96 - 2.59) | **<0.001*** |

BMI: body mass index; NR3C1: nuclear receptor subfamily 3 group C member 1; VLDL-c: very-low-density lipoprotein; LDL-c: low-density lipoprotein; CpG: cytosine-phosphate-guanine; IRR: incidence rate ratio; CI: confidence interval; p-value for univariate Poisson regression with robust variance, with a 5% significance level and metabolic syndrome (MetS) as the dependent variable; * p < 0.20, ** p <
